# Awareness and perceptions of managing recovery in psychiatric patients in a rehabilitation center: an observational study

**DOI:** 10.1192/j.eurpsy.2024.1487

**Published:** 2024-08-27

**Authors:** F. Franza, R. Croce, C. Esposito, I. Ferrara, R. Iannaccone, M. Imbimbo, M. Pizzano, S. Sanseverino, R. Silvestro, V. Vecchione

**Affiliations:** “Villa dei Pini” Clinic, Psychiatric Rehabilitation Centre, Avellino, Italy

## Abstract

**Introduction:**

In mental health prevention, person-centered, and rights-based approaches, the role of recovery is highlighted (WHO, 2021). Various evaluation tools are used in rehabilitation objectives and programs, including the Specific Levels of Functioning Scale (SLOF) (Mucci *et al*. Schizophr Res 2014;159 144-50) and the Recovery Assessment Scale – Domains and Stages (RAS-DS), a self-measure of mental health recovery. It includes 38 items clustered into four recovery domains and meets two functions. In addition to measuring self-reported outcomes, it increases service-user control towards objectives and recovery action plans (Honey et al. BMC Psychiatry 2023;23 500).

**Objectives:**

To evalue the efficacy of RAD-DS in a psychiatric rehabilitation facility to be used as a routine tool in daily rehabilitation activity.

**Methods:**

In our observational study, we recruited 103 inpatients (total: 103 patients, females: 38 patients, males: 65 patients) in a psychiatric rehabilitation facility. The patient presented with psychiatric disorders that met the diagnostic criteria of DMS-5 (schizophrenia, bipolar disorder, MDD, personality borderline disorder). Epidemiological data are shown in Table 1.

All patients were undergoing a psychiatric rehabilitation program and were observed during a one-year evaluation.

In all patients, the following rating scales were administered at baseline (T0) and after a year (T1):

For the evaluation of social measures, life outcomes, and functioning and recovery:

- Recovery Assessment Scale – Domains and Stages RAS-DS
- Specific Levels of Functioning Scale (SLOF)
- Global Assessment of Functioning (GAF)

For psychopathological evaluation:

- Brief Psychiatric Rating Scale (BPRS)

The data were statistically analyzed with the EZAnalyze 3.0 software for the Excel platform.

**Results:**

The RAS-DS total score results (Table 2) show a not significant difference between T0 vs. T1 (mean: 101.80 vs. 104.37, p. 0.193). An improvement in the score was observed after one year of rehabilitation treatment in the subgroup “*Doing things I value*” (T0 vs. T1: mean 16.15 vs. 18.77, p 0.001). Statistically significant differences were observed in the subgroups “*Mastering my illness*” (T0 vs. T1: mean 18.3 vs. 20.85, p. 0.021). In the other subgroups, the differences were not statistically significant. Interestingly, these results are comparable to those found with SLOF and GAF (respectively, p. 0.972 and p. 0.873).

**Image:**

**Figure:**
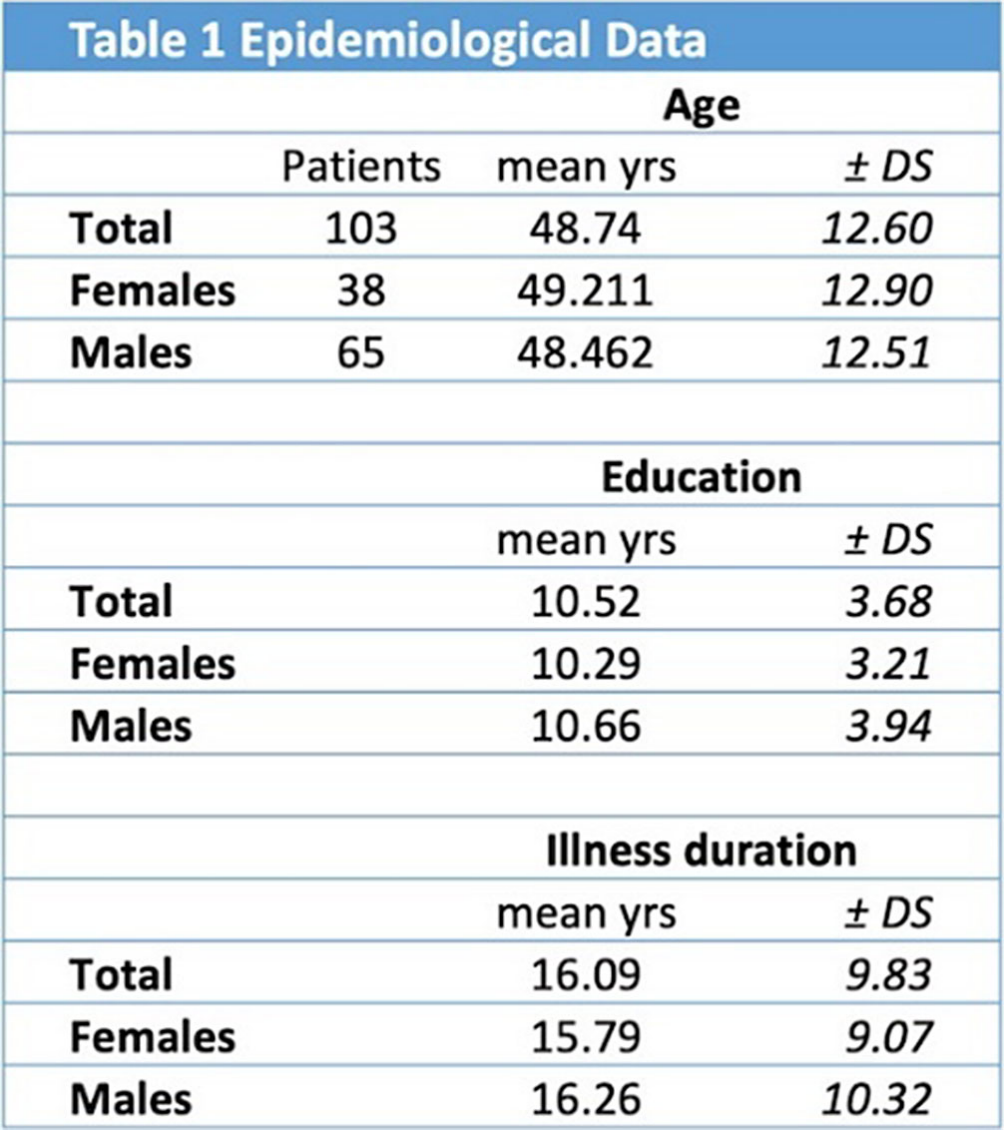

**Image 2:**

**Figure:**
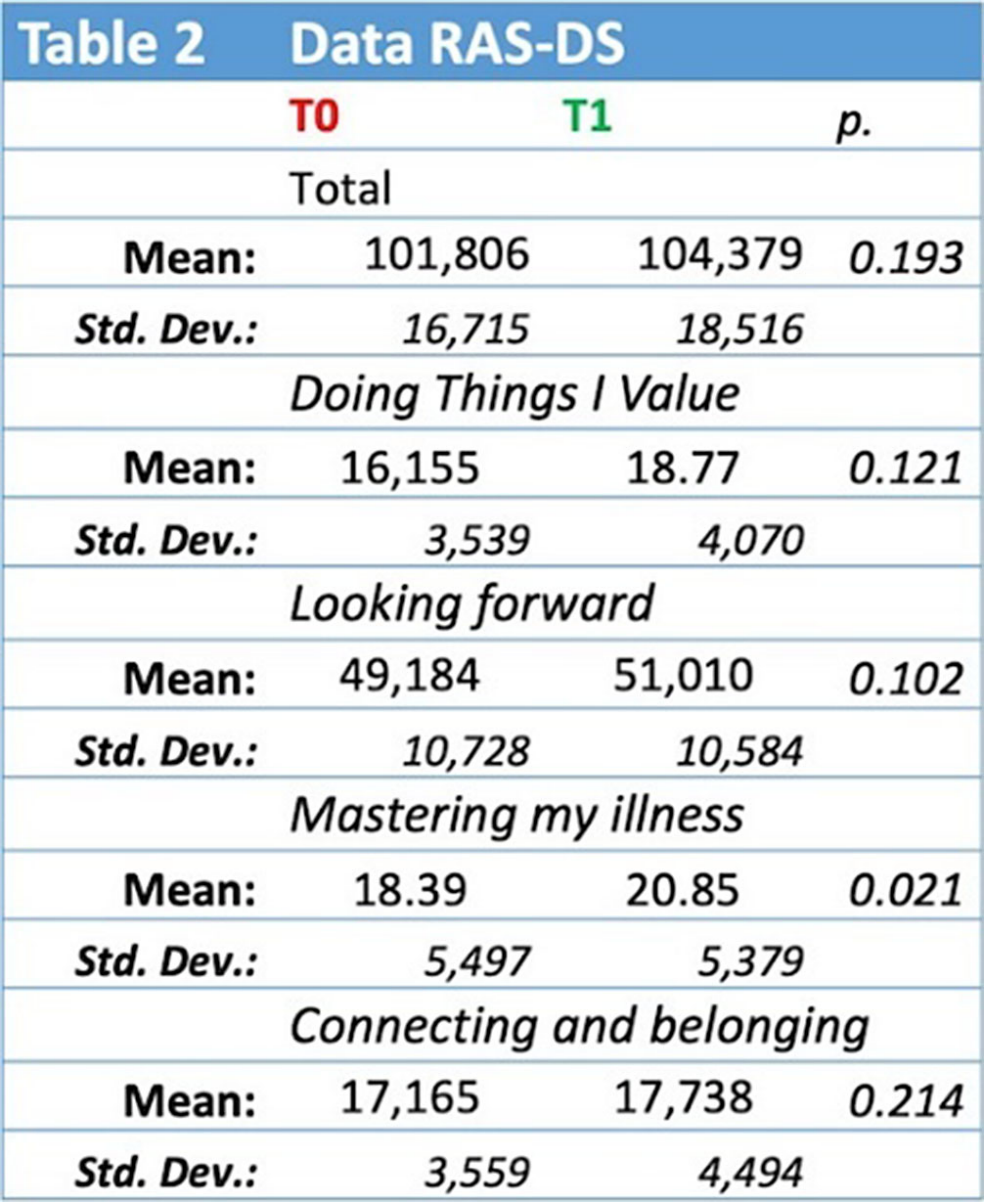

**Conclusions:**

The current trend of research and clinical practice is to give more importance to psychiatric rehabilitation treatment (Franza Psychiatr Danub 2022;34(Suppl 8) 9-13). The results obtained with our observational study indicate the possible usefulness of indicators of patient well-being, as well as the RAS-DS in the management of psychiatric rehabilitation programs. The expectations, indications, and perceptions of psychiatric patients can be decisive in improving recovery.

**Disclosure of Interest:**

None Declared

